# Effectiveness of the KC@H programme compared with clinic-based rehabilitation in patients recovering from ACL reconstruction: a study protocol for a single-centre, two-arm, single-blinded, randomised controlled superiority trial

**DOI:** 10.1136/bmjsem-2023-001868

**Published:** 2024-02-21

**Authors:** Joana Alegrete, Nuno Batalha, Orlando Fernandes, Jose Alberto Parraca, Ana Maria Rodrigues, Ana Rita Londral, João Paulo Sousa

**Affiliations:** 1 Department of Sport and Health, Universidade de Évora, Evora, Portugal; 2 Comprehensive Health Research Centre (CHRC), Universidade de Évora, Evora, Portugal; 3 NOVA Medical School, Universidade NOVA de Lisboa, Lisboa, Portugal; 4 NOVA School of Science and Technology, Universidade NOVA de Lisboa, Lisboa, Portugal; 5 Value for Health CoLAB, Universidade NOVA de Lisboa, Lisboa, Portugal

**Keywords:** Anterior cruciate ligament, Exercise rehabilitation, Knee ACL, Sports rehabilitation programs, Sports & exercise medicine

## Abstract

Patients who cannot fully comply with conventional clinic-based rehabilitation (CR) sessions after ACL reconstruction (ACLR) may find additional internet-based sessions beneficial. These remote sessions include therapeutic exercises that can be done at home, potentially extending the reach of rehabilitation services to underserved areas, prolonging the duration of care and providing improved supervision. The study’s main purpose is to determine if the Knee Care at Home (KC@H) programme is more effective than conventional CR alone in improving patient-reported, clinician-reported and physical functional performance outcome measures after ACLR. Additionally, the trial assesses the significance of changes in outcome measures for clinical practice.

This protocol outlines a randomised controlled trial for postoperative recovery following ACLR. Adult participants of both sexes who meet specific criteria will be randomly assigned to either the CR group or the KC@H group. Only the latter group will receive internet-based sessions of therapeutic exercises at home and CR sessions. A follow-up evaluation will be conducted for both groups 12 weeks after the intervention ends.

The trial protocol was approved by the Ethics Committee of the Universidade de Évora and complies with the Code of Ethics of the World Medical Association. All recordings will be stored on a secure server with limited access and deleted as soon as they are no longer needed.

The KC@H programme is expected to be superior to conventional CR for patients recovering from ACLR across multiple outcome measures. Also, the programme has the potential to promote superior recovery and extend the reach and duration of care.

Trial registration number: NCT05828355.

**WHAT IS ALREADY KNOWN ON THIS TOPIC**

- Communication technologies are being used for home-based rehabilitation after ACL reconstruction (ACLR). More data are needed to confirm their effectiveness compared with face-to-face rehabilitation.

**WHAT THIS STUDY ADDS**

- By demonstrating the effectiveness of the Knee Care at Home (KC@H) programme in maximising the recovery of patients after ACLR, we can offer healthcare professionals an innovative and complemental approach.

**HOW THIS STUDY MIGHT AFFECT RESEARCH, PRACTICE OR POLICY**

- The KC@H programme has the potential to expand the reach of rehabilitation services to underserved areas, prolong the duration of care and improve supervision using existing communication technologies.

## Background

Injuries to ACL can result in significant functional limitations.^1^ Thus, many physically active individuals often undergo ACL reconstruction (ACLR).^1^ Following surgery, individuals typically adhere to extensive and time-consuming clinic-based rehabilitation (CR) protocols essential for a complete recovery.^2 3^ These protocols involve executing therapeutic exercises guided by physiotherapists in CR sessions to alleviate knee pain, improve knee function and reduce the risk of further ACL injury.^3^

Postoperative progression in conventional CR stages requires support from standardised and evidence-based clinical guidelines. Currently, these guidelines are still being debated in the scientific literature.^3 4^ Progression is usually built on time-based principles, but experts have introduced criteria-based standards to improve the decision-making framework.^5^ As a result, conventional CR lacks consistency in its content and individual patient exposure.^4^ Each stage of recovery is distinct and relies on the patient’s expectations and progression over time.^4^

Due to each patient’s varying guidelines and progression criteria, physiotherapists face challenges in providing consistent care.^4^ This may contribute to some patients being unable to fully regain their pre-injury level of function and participate in strenuous activities.^3^

The ongoing debate surrounding evidence-based clinical guidelines is not the only concern regarding conventional CR protocols. There are also worries about the effectiveness of these protocols due to planning issues.^2^ Additionally, there is a significant gap between the completion of CR protocols and the return to normal activity levels, with some patients only attending a limited number of treatments and follow-up visits.^6^ When inadequate planning is combined with limited patient involvement, crucial clinical and functional outcomes can be disrupted.^7^

To address challenges in obtaining CR and enhance patient engagement, healthcare professionals recommend extended periods of personalised supervision tailored to patients’ specific needs and daily schedules.^4 7^ This increased supervision can potentially improve communication among healthcare professionals.^8^

Healthcare professionals can enhance patient involvement and engagement and reduce the previously described gap by incorporating internet-based interventions alongside CR.^8 9^ However, ensuring easy access to these interventions and integrating them with CR are crucial rather than using them in isolation.^9^

In recent years, several approaches have been using communication technologies to deliver rehabilitation services to individuals at their homes following ACLR.^10^ While some evidence suggests that these approaches may be equally beneficial as conventional CR, the available data are insufficient to support their effectiveness in patient- and clinician-reported outcomes fully.^11^

Internet-based interventions can potentially improve postoperative recovery by ensuring treatment continuity and providing patients with real-time feedback.^8 9^ This can be especially beneficial for those with difficulty accessing a rehabilitation clinic, as they can perform exercises at home.^12^

Additionally, it can reduce the need for travel and the cost of consultations.^8^ However, access to innovative approaches for ACLR recovery may be limited due to government organisations’ lack of prioritisation of orthopaedic services.^12^

The Knee Care at Home (KC@H) programme incorporated a new supplemental method to enhance conventional rehabilitation practises following ACLR. It includes an internet-based intervention with synchronous supervision of therapeutic exercises at home through videoconferencing software (remote sessions) and conventional CR (face-to-face sessions).

As such, this trial aims to determine whether the KC@H programme is more effective than conventional CR alone in improving patient-reported, clinician-reported and physical functional performance outcome measures after ACLR. Additionally, the trial assesses the significance of clinical changes in outcome measures for clinical practice.

## Methods

### Trial design

This protocol details the implementation of a randomised, controlled, parallel-group, two-arm, single-centre superiority trial. The allocation ratio is 1:1, and the protocol follows the Standard Protocol Items Recommendations for Interventional Trials Statement.^13^

**Figure 1:**
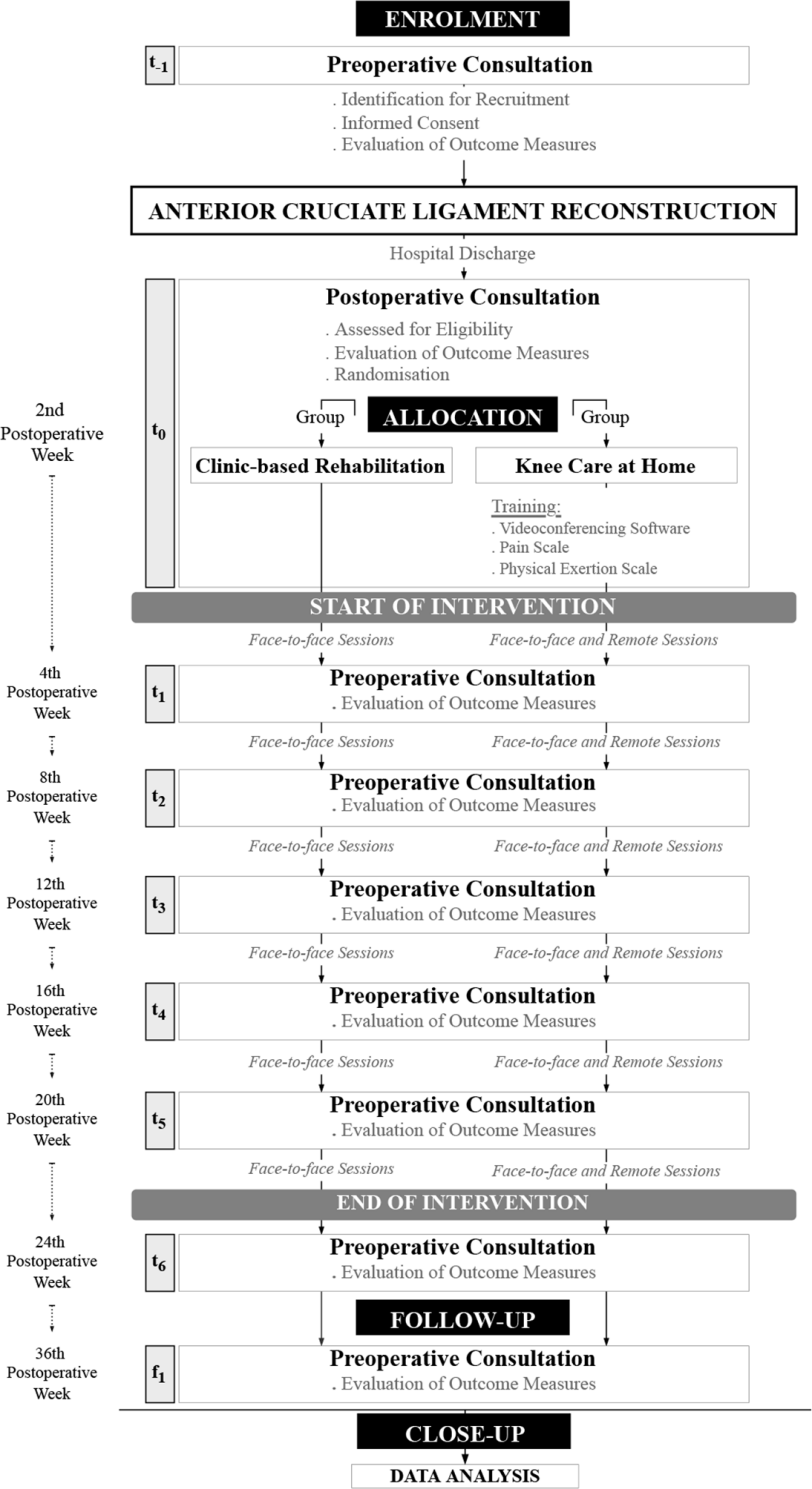
Flow chart of trial procedures.

A flow chart illustrates the trial procedures (figure 1). The study begins 2 weeks after hospital discharge and consists of a 22-week intervention period, followed by a 36-week follow-up. This protocol is being conducted simultaneously with a feasibility study.

#### Availability of data and materials

All records are kept on a secure server, and only the orthopaedic surgeon and the team’s principal investigator can access them. If any medical or research records need to be copied, the participant’s name and any other information will be removed. No personal information, such as name, address or telephone number, will leave the Hospital da Misericórdia de Évora (HME) database. All the information gathered for this investigation will be deleted or destroyed as soon as it is no longer needed.

### Sample selection and recruitment

Participants will be recruited at HME in Portugal. During their preoperative consultation with the orthopaedist, all patients with ACL rupture who are scheduled for ACLR at HME will be identified for recruitment. They will be given an informed consent form containing information on the trial protocol. Patients who agree to participate will receive a KC@H programme manual structured based on the Consensus on Exercise Reporting Template recommendations.^14^

### Study setting

Under the KC@H programme, a certified exercise and health coach will supervise synchronous remote sessions of therapeutic exercises at home, using internet-based videoconferencing software. Individualised face-to-face sessions with a physiotherapist will also be available for conventional CR participants at public or private rehabilitation clinics, depending on participant preference and availability. Outcome measures will be evaluated during scheduled consultations at the HME, except for pain intensity and physical exertion, which will be assessed during each remote session.

### Patient and public involvement statement

Patients and/or the public were not involved in the design, conduct, reporting, or dissemination plans of this research.

### Eligibility criteria

Adults of any sex who meet all eligibility criteria and provide written informed consent will be recruited for participation during the first postoperative consultation, which takes place 2 weeks after hospital discharge.

#### Inclusion criteria

1. Undergone primary ACLR regardless of surgical method and choice of autograft.
2. Aged between 18 and 55 years at the time of ACLR.
3. Have a healthy contralateral (opposite) knee.
4. The time between ACL injury and ACLR should not exceed 12 months.

#### Exclusion criteria

1. Declined to participate.
2. Concomitant osteochondral injuries.
3. Undergone multiple reconstructions of the lateral collateral ligament or posterior cruciate ligament.
4. Significant lower limb injuries within the 12 months before the ACL injury.
5. Medical conditions that may affect recovery.
6. Using medication for mental health disorders.
7. Severe impairments in communication or balance.

### Sample size calculation

The sample size was determined based on the Knee Injury and Osteoarthritis Outcome Score for pain.^15^ To have an 80% chance of detecting a significant increase in pain scores from 76.6 in the CR group to 92.7 in the KC@H group at the 5% level, approximately 40 patients are needed. A minimum sample size of 20 participants per group is required. Considering a 5% dropout rate in the control group and a 10% in the intervention group, the adjusted total sample size is 56 participants (28 participants per group).

### Group allocation

After confirming eligibility, participants will be randomly assigned (in a 1:1 ratio) to either the CR or KC@H group during the first postoperative consultation. Covariate adjustment will be conducted to ensure homogeneity in age and sex. Considering the covariates, the randomisation sequence will be tested using the ‘ralloc’ STATA module. The participants and the outcome assessor will be blinded to the allocation procedures.

A computer or tablet with an internet connection is required for those assigned to the KC@H group. Additional training will be provided for using the internet-based videoconferencing software, the Visual Analogue Scale (VAS) for pain^16^ and the Borg Category Ratio Scale (CR10) for physical exertion.^17^

### Blinding

Participants, orthopaedists and coaches will be informed about the group allocation during the trial, while the outcome assessor will remain blinded. The assessor will not be involved in any other aspects of the trial and will be explicitly instructed not to discuss group allocation with participants. Participants and orthopaedists will be instructed to refrain from discussing group allocation with the assessor.

### Trial retention

Retention strategies include participant trial identity cards (patient number only). Other strategies include emails or short messages sent by orthopaedists, exercise and health coaches during participant follow-up.

### Preoperative consultation

During the preoperative consultation, several activities are carried out. These include identifying potential participants for recruitment, obtaining informed consent, collecting sociodemographic data (such as age, sex assigned at birth, lower limb dominance, injury characteristics, mechanism of injury, time from injury to ACLR, smoking habits, education level and occupational status), assessing patient-reported outcomes (such as pain, symptoms, quality of life, swelling, catastrophising, anxiety, depression and stress), evaluating clinician-reported outcomes (such as height, weight, knee range of motion, thigh muscle length and thigh muscle strength) and providing training on videoconferencing software, pain scale and physical exertion scale.

### After surgery

Following ACLR, the surgical procedure details will be promptly documented in the participant’s medical record. Additionally, for the first 2 weeks after ACLR, participants will be sent a daily text message containing a link to an online questionnaire. This questionnaire is designed to assess pain intensity and pain management strategies.

### First postoperative consultation

Participants will have their first postoperative consultation 2 weeks after being discharged from the hospital. During this consultation, they will be assessed for eligibility and randomly assigned to either the CR or KC@H groups. An orthopaedist and a physiotherapist (assessor) will evaluate outcome measures at baseline.

### Intervention

The intervention begins immediately after the first postoperative consultation. Participants in both groups are advised to attend individual face-to-face sessions with a physiotherapist at a rehabilitation clinic and any postoperative consultation with the orthopaedist. We acknowledge that there may be accessibility issues in face-to-face sessions^7 9^ and variations in content and exposure^4^ that could impact our results. We randomly assigned both groups under comparable circumstances in our trial protocol to account for this. While we cannot control face-to-face sessions, we will monitor attendance and coverage weekly for both groups using a short messaging service. Additionally, we will strive to monitor the content similarity between face-to-face and remote sessions.

The intervention consists of internet-based synchronous remote sessions using videoconferencing software and face-to-face sessions. This enables participants to engage in therapeutic exercises at home under the guidance of an exercise and health coach. The remote sessions take place over 22 weeks, 3 days a week, lasting 40 min.

To provide a comprehensive description of the intervention, we have developed table 1 under the Template for Intervention Description and Replication recommendations.^18^

**Table 1:** Intervention description

Item	Comparison arm	Intervention arm
Brief name	Clinic-based rehabilitation	KC@H programme
Where	Rehabilitation clinic	Rehabilitation clinic and patient home
How	Face-to-face sessions	Face-to-face and remote sessions
Who	Physiotherapist	Physiotherapist and exercise and health coach
What	Dependent on rehabilitation clinic protocol and physiotherapist judgement	(face-to-face sessions) Dependent on rehabilitation clinic protocol and physiotherapist judgement. (remote sessions) KC@H manual providing general information, therapeutic exercises, time-based standards and criterion-based standards for progression
When	Starting from the second week after ACLR	Starting from the second week after ACLR
How much	Expected to last for 22 weeks, the number of sessions will be dependent on the patient’s preference and the availability of the rehabilitation clinic	(face-to-face sessions) Expected to last for 22 weeks, the number of sessions will depend on the patient’s preference and the availability of the rehabilitation clinic. (remote sessions) The programme will last for 22 weeks, with three sessions per week, each lasting 40 min. The exercise load will be monitored using the CR10 scale, while the pain intensity will be assessed using the VAS at the beginning and end of each session.
Tailoring/modifications	None	Time-based standards: immediate stage (0–2 weeks); early stage (2–4 weeks); intermediate stage (4–12 weeks); late stage (12–16 weeks); final stage (16–24 weeks) Criterion-based standards: inflammatory response; range of motion; lower limb muscle strength; gait; balance; core stability; agility; plyometrics

ACLR, ACL reconstruction; CR10, Borg Category Ratio Scale; KC@H, Knee Care at Home; VAS, Visual Analogue Scale.

#### KC@H programme manual

The KC@H manual describes all therapeutic exercises included in the programme, with patient progression determined based on objective criteria rather than a predetermined recovery timeline after ACLR.^19^ This approach, which combines time- and criterion-based standards, helps prevent adverse events during remote sessions and allows the intervention to be applied in various clinical scenarios.^19^

Due to the unavailability of specialised strength equipment for our remote sessions, we have used the CR10 scale to monitor training load and progress. For every exercise, we will record the duration (in minutes), number of sets and repetitions and the level of completion (complete, incomplete, unable to perform). Additionally, we will document the pain intensity at the start and end of each remote session. The orthopaedist will receive a detailed progress report containing all the information gathered during the remote sessions.

#### Postoperative phases

The postoperative period following ACLR lasts 24 weeks and is divided into five stages. The first stage, known as the ‘immediate postoperative’ stage, begins immediately after surgery and lasts 2 weeks. Although this stage starts before our trial intervention, the KC@H manual includes self-care recommendations developed by orthopaedic surgeons from HME. The baseline evaluation (t_0_) is conducted at the end of this stage, during the first postoperative consultation.

The second stage, which coincides with the start of our trial intervention, is called the ‘early postoperative’ stage. It begins immediately after our baseline evaluation and lasts 2 weeks until the next consultation with the orthopaedist (t_1_) in the fourth week after hospital discharge. This is the only intervention period that lasts less than 4 weeks.

After the early postoperative stage, the third stage, referred to as the ‘intermediate postoperative’ stage, is then divided into two substages, each lasting 4 weeks. At the end of each substage, a consultation with the orthopaedist and subsequent evaluation occurs at the 8th (t_2_) and 12th (t_3_) weeks.

The fourth stage, ‘late postoperative’, begins immediately after t_3_ and continues for 4 weeks. At week 16, another consultation with the orthopaedist (t_4_) is scheduled. The final stage, ‘final postoperative’, is divided into two substages, each lasting 4 weeks. Following t_4_, a consultation with the orthopaedist and subsequent evaluation occurs on the 20th (t_5_) and 24th (t_6_) weeks after hospital discharge. The 24th-week consultation marks the end of the intervention.

Additionally, patients in both groups will be invited for a scheduled follow-up at the end of the postoperative period, approximately at week 36 (9 months) after hospital discharge and 12 weeks (3 months) after the conclusion of the trial intervention (f_1_).

#### Outcomes measures and assessments

Outcome measures will be assessed during scheduled consultations with the orthopaedist. The exercise and health coach will record pain intensity and physical exertion during each remote session. A physiotherapist (assessor) will remain consistent for every evaluation and conduct assessments during consultations with the orthopaedist.

The outcome measures and assessment tools for both groups are categorised into patient-reported (table 2), clinician-reported (table 3), physical functional performance and clinical relevance (table 4). These tables outline the procedures for each outcome measure and the tools used in the evaluations.

**Table 2:** Patient-reported outcome measures

Outcome	Assessment tool	Procedures
Knee pain during the last 24 hours	Visual Analogue Scale^16^	Patients provide feedback on knee pain intensity by marking a point on a 100 mm straight horizontal line. The beginning of the line represents ‘no pain’, while the end represents the ‘worst possible pain’. A ruler measures the distance (millimetres) from 0 to the patient’s marking. A numerical value and Wong-Baker faces scale will also be presented to the patient for reference.
Knee pain during the last week	Knee Injury and Osteoarthritis Outcome Score (KOOS)^22^	Patients provide feedback on the severity of their knee pain using a standard 5-point Likert scale. Each response option is assigned a numerical value (0 for none, 1 for mild, 2 for moderate, 3 for severe and 4 for extreme). A score of 0 indicates no pain, while a score of 32 (based on 8 questions) indicates extreme pain.
Knee symptoms and stiffness during the last week	KOOS^22^	Patients are asked to rate their knee symptoms and stiffness using a standard 5-point Likert scale, with each response option assigned a numerical value (0, 1, 2, 3, 4). A score of 0 indicates total absence of symptoms, while a score of 20 (based on 5 questions) indicates constant symptoms. For knee stiffness, a score of 0 indicates total absence, while a score of 8 (based on 2 questions) indicates extreme stiffness.
Knee-related physical function during the last week	Knee Injury and Osteoarthritis Outcome Score—Physical Function Short Form^23^	Patients use a standard 5-point Likert scale to provide feedback on their knee-related physical function. Each response option is assigned a numerical value (0 for none, 1 for mild, 2 for moderate, 3 for severe and 4 for extreme). A score of 0 indicates no difficulties, while a 28 (based on 7 questions) indicates extreme difficulties.
Knee function (daily living, sports and recreational activities) during the last week	KOOS^22^	Patients are asked to rate their knee function in daily living, sports and recreational activities using a 5-point Likert scale. Each response option is given a numerical value (0 for none, 1 for mild, 2 for moderate, 3 for severe and 4 for extreme). For knee function in daily living, a score of 0 indicates no difficulties, while a score of 68 (based on 17 questions) indicates extreme difficulties. Similarly, for knee function in sports and recreational activities, a score of 0 indicates no difficulties, while a score of 20 (based on 5 questions) indicates extreme difficulties.
Knee-related quality of life during the last week	KOOS^22^	Patients using a standard 5-point Likert scale to provide feedback on their knee-related quality of life. Each response option is given a numerical value (0, 1, 2, 3, 4), and a raw score is determined based on their responses. A score of 0 indicates no impact, while a score of 4 (based on 4 questions) indicates extreme impact on the quality of life.
Pain catastrophising	Pain Catastrophizing Scale^24^	Patients are asked to provide feedback on their thoughts, feelings or perceptions related to pain using a standard 5-point Likert scale. Each response option is assigned a numerical value (0 for ‘not at all’, 1 for ‘a slight degree’, 2 for ‘moderate degree’, 3 for ‘great degree’ and 4 for ‘all the time’). A score of 0 indicates a total absence of catastrophic thinking, while a score of 52 (based on 13 questions) indicates a persistent tendency towards catastrophising thoughts about pain.
Anxiety, depression and stress during last week	Depression Anxiety Stress Scales (DASS-21) short-form^25^	Patients use a standard 4-point Likert scale to report their levels of depression, anxiety and stress. Each response option is assigned a numerical value (0 for ‘did not apply to me at all’, 1 for ‘applied to me to some degree or some of the time’, 2 for ‘applied to me to a considerable degree or a good part of the time’ and 3 for ‘applied to me very much or most of the time’). Cut-off scores are used to determine the severity of the patient’s condition. For depression, based on 7 items, scores of 0 to 9 are considered normal, 10 to 13 are mild, 14 to 20 are moderate, 21 to 27 are severe and 28 or higher are extremely severe. For anxiety, based on 7 items, scores of 0 to 7 are normal, 8 to 9 are mild, 10 to 14 are moderate, 15 to 19 are severe and 20 or higher are extremely severe. Based on 7 items for stress, scores of 0 to 14 are normal, 15 to 18 are mild, 19 to 25 are moderate, 26 to 33 are severe and 34 or higher are extremely severe.

**Table 3:** Clinician-reported outcome measures

Outcome	Assessment tool	Procedures
Height	Stadiometer	Patients are measured for standing height in centimetres using a stadiometer placed against the wall. They are instructed to stand with their backs against the wall, heels together, head in a natural position, legs straight, arms at their sides and shoulders relaxed. They should not wear shoes or heavy clothing that could affect their height measurement. The measurement arm is lowered to rest gently on the patient’s head, and the measurement is rounded off to the nearest decimal.
Weight (body mass)	Analogue Scale	A traditional weighing scale assesses patients for weight (body mass in kilograms). The scale is placed on a flat and stable surface, and patients are advised not to wear shoes or heavy clothing that could affect their weight measurement. They are instructed to stand still in the centre of the scale, with their feet evenly positioned and their weight evenly distributed. The weight measurement is then recorded and rounded off to the nearest decimal.
Knee joint effusion	Patellar Tap/Ballottement Test^26^	Patients are evaluated for knee effusion while lying supine on an examination table. The assessor applies downward strokes from the thigh to the leg with the non-dominant hand and then grasps the upper portion of the knee just above the patella. Using two fingers of the dominant hand, they press the patella against the femur in a posterior direction. Increased patellar waving or a spongy joint sensation indicates a positive test result for knee effusion. The test is then repeated on the opposite knee for comparison.
Passive knee flexion and extension range of motion	Hand-held Goniometry (HHG)^27^	Passive knee extension and flexion range of motion are measured in patients using a long-arm goniometer. Both knees are measured for comparison with the goniometer axis placed at the lateral epicondyle of the femur. The proximal arm of the goniometer is placed alongside the lateral midline of the femur, using the greater trochanter as a reference. In contrast, the distal arm is placed alongside the lateral midline of the fibula, using the lateral malleolus and fibular head as references. For passive knee flexion, the patient lies supine on the examination table, with the lower limbs in an anatomical position. The assessor flexes the patient’s knee through the available range of motion by sliding the patient’s foot along the table towards the pelvis and then taking the passive range of motion measurement. For passive knee extension, the patient lies in a supine position on the examination table with the lower limbs in an anatomical position and a towel under the ankle with the knee extended as far as possible. The assessor provides added pressure to the knee in the direction of extension and then takes the passive range of motion measurement.
Knee extensor and flexor muscle length	HHG^27^	The length of the knee flexors and knee extensors muscles in patients is measured using specific tests and a long-arm goniometer. The measurements are taken on both knees for comparison, with the goniometer axis placed at the lateral epicondyle of the femur. For the knee flexors muscle length (Thomas test), the patient lies in a supine position with the hip of the lower extremity to be measured and extended on the examination table. The leg is supported on the table, and the knee is just past the table’s edge (test side). The patient is asked to flex the contralateral hip and grasp the knee to the chest until the lumbar spine is felt to flatten against the table, and the measurement of knee flexion is taken (test side). For the knee flexors muscle length (prone technique), the patient lies in a prone position with both hips of the lower extremity extended on the examination table. The knee to be tested moves to maximal achieved flexion, and the measurement of knee flexion is taken (test side). For the knee extensors muscle length (distal hamstring length test), the patient lies in a supine position with the hip of the lower extremity to be measured in 90 degrees of flexion on the examination table. While the assessor helps maintain the hip position, the patient executes a knee extension, and the knee flexion is measured (test side).
Extensor and flexor isometric muscle strength	Hand-held Dynamometry^28^	The microFET2 is used to measure the isometric strength of the hamstrings and quadriceps in patients. This measurement is taken on both knees to allow for comparison. The patient is seated on an examination table with their hips and knees flexed at a 90-degree angle. The assessor stabilises the patient’s leg using the leg of the examination table as support for the hand-held dynamometer. The patient is then instructed to apply maximum force against the device. First, the patient is asked to try to extend the knee, and then they are asked to flex the knee. Three repetitions are performed for both extension and flexion.

**Table 4:** Physical functional performance and clinical relevance outcome measures

Outcome	Assessment tool	Procedures
Physical functional performance		
Hop distance (single Leg)	Single Leg Hop for Distance Test^29^	Patients are instructed to stand on the leg being tested, hop and then land on the same limb, jumping as far as possible. The distance hopped is measured at the level of the great toe and recorded to the nearest centimetre using a standard measuring tape fixed to the floor. Both lower limbs are evaluated for comparison.
Stairs (ascent/descent)	Stair Climbing Test^30^	Patients will be timed as they ascend and descend a flight of 12 stairs as quickly as possible. The number of steps they perform in 30-second intervals will also be counted.
Clinical relevance		
Symptom state	Patient Acceptable Symptomatic State^21^	Patients provide their perspectives on the final Patient Reported Outcome Measures (PROM) scores, which indicate their satisfaction with symptoms. For each PROM score, patients are asked a question with the answer option ‘yes’ or ‘no’.
Clinical benefit	Substantial Clinical Benefit^21^	Patients are surveyed to gather their perspective on the change in PROM scores that indicates a significant or optimal improvement. For each change in score, patients are asked a question with answer options displayed on a 7-point Likert scale, ranging from ‘significant and marked improvement’ to ‘no improvement at all’.
Clinical difference	Minimal Clinically Important Difference (MCID)^21^	The patients will be asked about their opinion on the minimum change in PROM scores that would significantly improve their condition. We will use an anchor-based technique to determine the MCID.

Postoperative evaluations for patients in both study groups will be scheduled at the following intervals after hospital discharge: (t_0_) 2 weeks; (t_1_) 4 weeks; (t_2_) 8 weeks; (t_3_) 12 weeks; (t_4_) 16 weeks; (t_5_) 20 weeks; (t_6_) 24 weeks, coinciding with the end of surgery; and (f_1_) 36 weeks for follow-up. A detailed schedule of all evaluations and collected outcome measures can be found in table 5.

**Table 5:** Evaluations and outcomes measures schedule

	Preoperative	Postoperative							Follow-up
		2nd week	4th week	8th week	12th week	16th week	20nd week	24th week	36th week
	Consultation (t_−1_)	Consultation (t_0_)	Consultation (t_1_)	Consultation (t_2_)	Consultation (t_3_)	Consultation (t_4_)	Consultation (t_5_)	Consultation (t_6_)	Consultation (f_1_)
Enrolment									
Prescreening/informed consent	x								
Eligibility		x							
Allocation		x							
Interventions									
Clinic-based rehabilitation (face-to-face sessions)		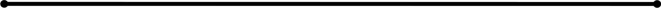							
Knee Care at Home (face-to-face and remote sessions)		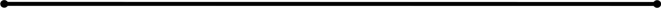							
Outcome measures									
Patient-reported									
Pain	x	x	x	x	x	x	x	x	x
Pain catastrophising	x	x	x	x	x	x	x	x	x
Symptoms	x	x	x	x	x	x	x	x	x
Function	x	x	x	x	x	x	x	x	x
Quality of life	x	x	x	x	x	x	x	x	x
Anxiety/depression/stress	x	x	x	x	x	x	x	x	x
Clinician-reported									
Height	x	x	x	x	x	x	x	x	x
Mass (weight)	x	x	x	x	x	x	x	x	x
Joint effusion		x	x	x	x	x	x	x	x
Range of motion		x	x	x	x	x	x	x	x
Muscle length		x	x	x	x	x	x	x	x
Muscle strength		x	x	x	x	x	x	x	x
Physical functional performance									
Hop distance (single leg)						x	x	x	x
Stair (ascent/descent)		x*	x*	x*	x	x	x	x	x
Clinical relevance									
Symptom state		x	x	x	x	x	x	x	x
Clinical benefit		x	x	x	x	x	x	x	x
Clinical difference		x	x	x	x	x	x	x	x

### Data management

The orthopaedist and lead investigator will oversee data collection, development and security. Complete data analysis will be conducted after the trial protocol. The orthopaedist will manage access to clinical records stored in HME’s secure database and only disclose the study’s relevant information. Any necessary pictures will be encrypted for protection and promptly deleted after the trial concludes. All participant data will be linked to a logged patient number inputted into the analysis software along with other relevant data, and all spreadsheets will be encrypted.

The Limb Symmetry Index will calculate the range of motion, muscle length, muscle strength and hop performance by comparing the involved and uninvolved sides and normalising for mass and height.^4^ Additionally, physical functional performance testing will be supplemented with data from the CR10 scale and VAS for pain.

### Statistical analysis

The strength of evidence for the applied methods will be measured using a significance level of 5%. The analysis will adhere to intention-to-treat principles, and patient characteristics will be presented using descriptive statistical tests. The chosen method for examining outcomes will account for within-subject correlations in repeated measures analysis of variance.^20^ To assess the effect of treatment, between-group differences will be calculated by constructing linear mixed models with interaction terms of treatment groups versus time. All models will be adjusted for baseline estimates.

The Minimal Clinically Important Difference (MCID) will be determined using an anchor-based or distribution-based method if the former is unsuitable. The anchor-based technique will use the receiver operating characteristic (ROC) curve to identify the optimal cut-off point for distinguishing between groups with minimal and no change. The cut-off value will be determined by maximising specificity and sensitivity. The area under the ROC curve will be used to assess reliability, with acceptable values falling between 0.70 and 0.80 and excellent values ranging from 0.80 to 0.90. The distribution-based method will rely on SD and effect size-based estimation. Across various studies, an MCID corresponds to an SD value of 0.5, and to calculate the change MCID, the SD of the baseline scores is multiplied by 0.2 (small effect size).^21^

All statistical analysis and presentation will adhere to the recommendations outlined in CHecklist for statistical Assessment of Medical Papers: the CHAMP.^20^

### Benefits and harms

While benefits from the trial are not guaranteed, significant adverse effects are not expected. Participants may experience increased discomfort after exercising, a common side effect when starting a new activity. The KC@H programme is insured to provide coverage for participants in the event of any damage.

The KC@H programme carries a potential risk of adverse events, which will be minimised through precautionary measures. Participants are encouraged to contact the lead investigator using the provided phone number for guidance or in case of any adverse events or questions. All adverse effects will be documented and reported to the Institutional Review Boards of the Universidade de Évora and the HME.

## Discussion

The research findings are expected to demonstrate the KC@H programme is more effective than conventional CR alone for patients recovering from ACLR. The KC@H programme supplements conventional CR with internet-based remote sessions. Using videoconferencing software, these remote sessions are guided by exercise and health professionals, allowing patients to complete a set of therapeutic exercises in the comfort of their homes. The KC@H programme also allows for close monitoring of pain intensity, exercise load and progression in postoperative stages. This progression not only observes time-based principles but also objective criteria-based standards. Additionally, using KC@H remote sessions may help healthcare professionals to ensure more equal patient outcomes.

Our ambition was to craft an innovative pathway for the rehabilitation of ACLR that is convenient and productive for both patients and healthcare professionals. By combining conventional CR and the KC@H programme remote sessions, the reach of ACL rehabilitation services can be expanded, especially for those in underserved areas. Using commonly available communication technologies in the KC@H programme increases the likelihood of easily implementing this delivery method.

We will take several steps to ensure the internal and external validity of our findings. Participants will be selected based on specific but not overly restrictive criteria to ensure they represent the target population. While recruitment will occur in a single hospital unit, it serves a large geographical area. Standardised processes will be implemented to ensure the trial protocol is followed and the collected data are high quality. Suppose our findings show that the KC@H programme is more effective than CR alone in improving patient-reported, clinician-reported and physical functional performance outcome measures after ACLR. In that case, it may lead to improved functional recovery and provide healthcare professionals with a new complementary approach to rehabilitation services. We aim to assist healthcare professionals in searching for innovative strategies to improve their rehabilitation practice following ACLR and to stimulate the development of further research in telerehabilitation.

### Trial status

The trial protocol has been submitted before the start of the recruitment.

### Auditing

JPS and NB will monitor the trial’s progress every 4 weeks to ensure that all phases of the protocol are being executed as intended.

### Dissemination policy

This protocol will be published in scientific journals and presented at conferences. All researchers who contribute to the protocol's development, implementation or analysis will be recognised as authors in the resulting publications.

### Confidentiality

This document is confidential and is the property of the Department of Sports and Health of the Universidade de Évora and the Comprehensive Health Research Centre. No part of it may be transmitted, reproduced, published or used without prior written authorisation from the institutions.

## Data Availability

No data are available.
